# Bevacizumab with Chemotherapy as a First-Line Treatment for Advanced Ovarian Cancer in a Serbian Cohort

**DOI:** 10.3390/medicina58050607

**Published:** 2022-04-27

**Authors:** Irena Conic, Bojan Nedovic, Slavica Stojnev, Ilinka Todorovska, Aleksandra Dimitrijevic, Miljan Krstic, Ivana Djordjevic, Biljana Djordjevic

**Affiliations:** 1 Faculty of Medicine, University of Nis, 18000 Nis, Serbia; nedovicbn@gmail.com (B.N.); slavicastojnev@gmail.com (S.S.); aleksdimitrijevic93@gmail.com (A.D.); krstic.miljan@gmail.com (M.K.); ivanadjordjevic01.12@gmail.com (I.D.); biljanadjordjevic997@gmail.com (B.D.); 2 Oncology Clinic, Clinical Center Nis, 18000 Nis, Serbia; ilinkatod@gmail.com; 3 Center for Pathology, Clinical Center Nis, 18000 Nis, Serbia

**Keywords:** bevacizumab, ovarian cancer, progression-free survival, overall survival

## Abstract

*Background and Objectives*: For stage IIIb–IV ovarian cancer, bevacizumab-containing treatment is considered the standard of care. The purpose of this study was to evaluate the efficacy of bevacizumab in combination with carboplatin and paclitaxel as a first-line treatment for advanced ovarian cancer. *Materials and Methods*: Eligible patients had stage IIIc–IV ovarian cancer according to the International Federation of Gynecology and Obstetrics with no clinical signs or symptoms of gastrointestinal obstruction or a history of abdominal fistulae, gastrointestinal perforation, or intra-abdominal abscess or evidence of rectosigmoid involvement by pelvic examination, bowel involvement on computed tomography, or clinical symptoms of bowel obstruction in the previous 6 months. After debulking surgery, the patients received 175 mg/m^2^ paclitaxel and carboplatin (AUC 6) for the first six cycles and 7.5 mg/kg bevacizumab every three weeks up to 17 cycles until disease progression, unacceptable toxicity, or consent withdrawal. The primary endpoint was progression-free survival. The secondary endpoint was overall survival. *Results*: Between April 2017 and March 2020, 35 patients began study treatment. Bevacizumab was administered at 7.5 mg/kg in all the patients and for more than 7.5 months in 70% of them. The median progression-free survival was 20 months (95% CI: 16–23). The median overall survival was not reached. *Conclusions*: This was, to our knowledge, the first trial in Serbia to show progression-free survival and overall survival of combination regimens in advanced ovarian cancer. Based on the observed progression-free survival, bevacizumab combined with chemotherapy should be considered as a standard option in advanced ovarian cancer.

## 1. Introduction

Ovarian cancer (OC) is the eighth most prevalent cancer in women and the eighteenth most prevalent cancer in the general population. In 2018, over 300,000 new cases were reported; Serbia had the highest rate of ovarian cancer (the age-standardized rate was 16.6 per 100,000) [1]. Most women feature advanced ovarian cancer at the time of diagnosis, which accounts for the high fatality rate [2]. Furthermore, the majority of patients relapse following the standard therapy with debulking surgery and platinum-based chemotherapy [3].

Modulation of the vascular endothelial growth factor (VEGF) has progressed from a theoretical notion to an essential component of therapy [4]. In two large-scale phase 3 randomized trials, GOG-218 and ICON7, in first-line settings, the addition of the anti-VEGF monoclonal antibody bevacizumab to routinely delivered carboplatin and paclitaxel chemotherapy significantly improved progression-free survival [5,6]. For platinum-sensitive recurrent and platinum-resistant recurrent epithelial ovarian cancer, bevacizumab is regarded as the standard-of-care front-line treatment [7]. In advanced and recurrent epithelial ovarian cancer, bevacizumab-containing therapies show a significant progression-free survival (PFS) benefit, and although not associated with improved survival in the first-line setting, improved overall survival did reach statistical significance in the recurrent setting [8].

To our knowledge, this study was the first to investigate the effects of bevacizumab as a standardized treatment for advanced ovarian cancer in patients in Serbia. The effect on the overall survival as well as progression-free survival must be understood in order to thoroughly assess a new treatment. We report the median progression-free survival (PFS), the Q1 overall survival (OS), and the clinical characteristics of the patients with stage IIIc, IVa, and IVb advanced ovarian cancer (according to the International Federation of Gynecology and Obstetrics (FIGO)) who initially underwent total abdominal hysterectomy with bilateral salpingo-oophorectomy and omentectomy.

## 2. Materials and Methods

We recruited study participants among patients admitted to the oncology clinic of the University Clinical Center of Nis (Serbia) between April 2017 and March 2020. The eligible patients had histologically confirmed epithelial ovarian cancer [9,10]. All the patients initially underwent total abdominal hysterectomy with bilateral salpingo-oophorectomy and omentectomy. The study required participants to be at least 18 years old, have an Eastern Cooperative Oncology Group performance status (ECOG) of 0 or 1, and have sufficient liver, kidney, and bone marrow function. The FIGO stage was established at the time of the first diagnosis, and only patients with FIGO stages IIIc and IV were included. We excluded the patients with a history of bowel obstruction (including subocclusive disease) because of the underlying disease, with an abdominal fistula, gastrointestinal (GI) perforation, or intra-abdominal abscess, or with evidence of rectosigmoid involvement by pelvic examination or colonoscopy, bowel involvement on computed tomography, or clinical symptoms of bowel obstruction. Furthermore, the patients with a history or evidence of thrombotic or hemorrhagic diseases within 6 months of the start of the research, uncontrolled hypertension or current clinically significant cardiovascular disease, or nonhealing wounds or ulcers were excluded.

For the patients with measurable and non-measurable disease, progression was defined by RECIST (version 1.1) [11]. Every effort was made to document the progression even after discontinuation of the treatment.

The recruited patients received 175 mg/m^2^ paclitaxel and carboplatin (AUC 6) for the first six cycles and 7.5 mg/kg bevacizumab every three weeks up to 17 cycles until disease progression, unacceptable toxicity, or consent withdrawal. It was not possible to lower the dose of bevacizumab. The chemotherapy dose adjustment recommendations were in line with the current clinical practice.

The same assessment technique was used to assess the tumor at baseline and every nine weeks during the trial (computed tomography or magnetic resonance imaging in the case of contrast allergy). We reviewed safety data actively. Before each cycle and within 30 days of finishing the therapy, safety was examined. Adverse events were graded using the National Cancer Institute’s Common Terminology Criteria for Adverse Events (version 5.0). Selected clinical and pathological features were reviewed: histological subtype, ECOG performance status stage, FIGO stage, ovarian cancer grade, pleural effusion, and ascites. Before undergoing any study-specific procedures, all the patients submitted written informed consent forms. The research was conducted under the principles of the Declaration of Helsinki, as well as those specified in the Tripartite Guideline for Good Clinical Practice of the International Conference on Harmonization and the EU Clinical Trial Directive.

The primary endpoint was progression-free survival calculated from the date of surgery to the date of RECIST 1.1 progression. The time from assignment to death as a consequence of any cause was defined as the overall survival, and the patients who were alive at the time of the analysis were censored at the date of the last contact. The patients were observed for survival until July 2020. Progression-free survival and the overall survival were estimated using the Kaplan-Meier methodology. All the analyses were carried out with Stata software package release 17.

## 3. Results

Between April 2017 and March 2020, 35 patients were enrolled. Over 94% of the patients had serous adenocarcinoma, with the majority of malignancies being high-grade (88.57%). The cohort had a dismal prognosis: 54% had FIGO stage IIIc cancer with a maximal residual lesion diameter of more than 1 cm and 46% had stage IV cancer. We summarized the clinical features in Table 1.

**Figure 1:**
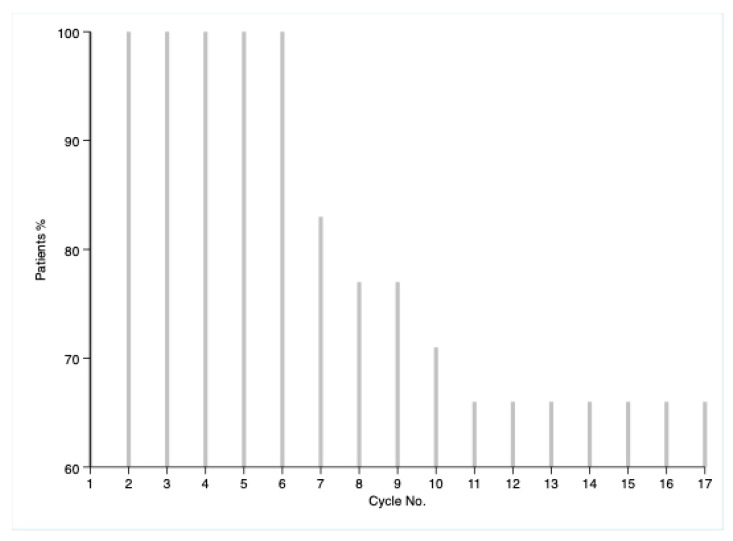
Treatment exposure summary.

Sixty-five percent of the patients completed the planned treatment. Thirty-five percent of the trial participants dropped out early, with disease progression being the most prevalent cause. We report the treatment exposure summary, with one cycle being 3 weeks long, in Figure 1.

**Figure 2:**
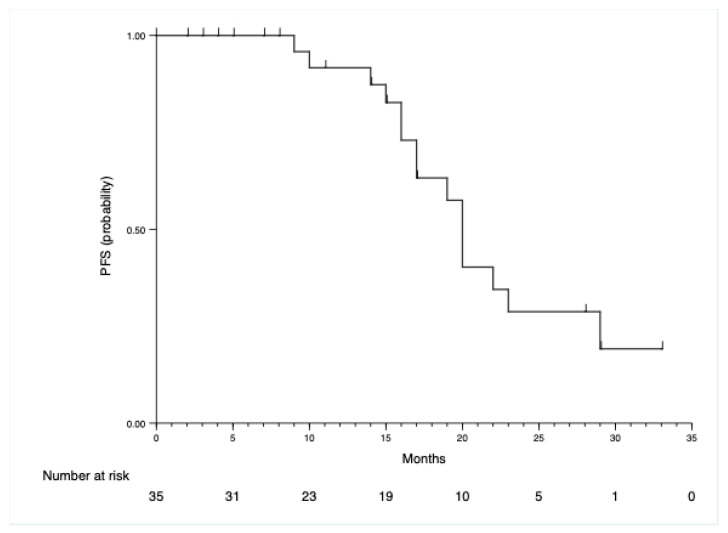
Kaplan-Meier estimates of progression-free survival (PFS) based on investigator assessment.

The median progression-free survival was 20 months (95% CI: 16–23) (Figure 2).

**Figure 3:**
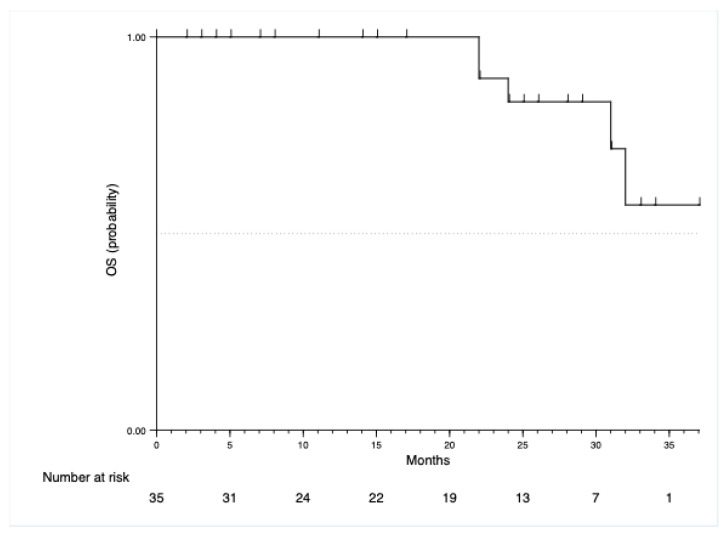
Kaplan-Meier estimates of progression-free survival (PFS) based on investigator assessment.

The median follow-up was 28 months. The median overall survival was not reached; we can report the first quartile of 31 months (Figure 3).

Subgroup analysis for the age of progression-free survival and the overall survival is reported in Table 2.

The median overall survival in the group of patients ≥65 years old was 32 months. Before beginning the study medication, all the 13 patients with ascites at baseline underwent paracentesis. With ascites, the median progression-free survival was 19 months (from 16 in Q1 to 29 in Q3). Adverse events of specific interest occurred in just two patients (hemorrhage in the corpus vitreum, deep vein thrombosis). There were no deaths throughout the research period that were not primarily determined by progressing illness.

## 4. Discussion

Our study met its primary goal, demonstrating a 20-month progression-free survival when bevacizumab was added to chemotherapy for advanced ovarian cancer. The observed overall survival for Q1 was 31 months (the secondary endpoint median overall survival not met). To our knowledge, this was the first study in Serbia to show PFS and the OS with combination therapy in this setting.

Findings of our study supplement the previously published research [6,12,13,14,15]. In the first-line setting, bevacizumab combined with chemotherapy significantly improved PFS compared with chemotherapy alone. Nevertheless, its effectiveness is still uncertain in individuals whose illness relapses after receiving a first-line bevacizumab-containing treatment [16]. Reported in clear cell carcinoma patients, continuous angiogenesis inhibition by bevacizumab may lead to bevacizumab maintenance’s improved efficacy beyond progression [17]. Furthermore, bevacizumab improves the outcome in metastatic colorectal cancer patients when administered with the second-line chemotherapy following initial progression (mCRC) [18]. In patients receiving double the dose of bevacizumab following the first disease progression, Ca˘inap et al. discovered that increasing the bevacizumab dose intensity could outweigh the prognostic influence of the primary tumor location [19]. However, a recent study found that second-line chemotherapy had poor clinical results in metastatic colorectal cancer patients (mCRC) who progressed early after the first-line treatment regardless of the use of antiangiogenic drugs [20].

Preliminary evidence is not conclusive that combining immuno-oncology agents, an antiangiogenic agent, and a poly(ADP)-ribose polymerase (PARP) inhibitor (triplet treatment) may result in a synergistic anticancer impact [5,21,22]. The addition of bevacizumab in our study resulted in a better management of ascites.

According to a recent study of ascites in patients with chemotherapy-resistant epithelial ovarian cancer, the median paracentesis-free interval was 4.29-fold (95% CI: 2.4–5.8) higher after the first dose of intraperitoneal bevacizumab compared to the time between paracenteses before the study entry [23].

Wichelmann et al. investigated and detailed the post-market incidents of bevacizumab-induced gastrointestinal perforation reported to the Food and Drug Administration’s Adverse Event Reporting System (FAERS) database [24]. Our study had strong exclusion procedures in place to guarantee that patients who were at high risk of GI perforation were not enrolled. GI perforation incidence was effectively reduced by this method. Before the tolerance reported in our study can be extended to others, more research is needed. Carefully outlined radiologic criteria may help identify individuals at most risk of GI perforation in heavily pretreated patients.

Our study had limitations, including a lack of specimen collection for biomarker analyses and quality-of-life data. However, the strengths lie in careful adherence to the RECIST 1.1-defined progression and evaluation schedule.

## 5. Conclusions

In conclusion, ours was the first study, to our knowledge, in Serbia to show PFS and the OS of a combination regimen in advanced ovarian cancer. Based on the observed PFS, we should account for bevacizumab with chemotherapy as a conventional approach in advanced ovarian cancer based on the observed PFS.

## Figures and Tables

**Table 1:** Baseline clinical features.

Characteristic	N = 35	Percentage
Age, years		
Mean, SD	61.8, 10.18	
Histological subtype		
Serous	33	94.29
Endometrioid	0	0
Mucinous	1	2.86
Clear cell	1	2.86
ECOG performance status		
0	10	28.57
1	25	71.43
FIGO stage		
IIIc	19	54.29
IVa	12	34.29
IVb	4	11.43
Ovarian cancer grade		
Low grade	4	11.43
High grade	31	88.57
Ascites		
Yes	13	37.14
No	22	62.86

**Table 2:** Subgroup analysis for the age of progression-free survival (PFS) and the overall survival (OS).

Subgroup	PFS, No. of Events/No. of Patients	PFS, Median	OS, No. of Events/No. of Patients	OS, Median
Age, years				
<65	7/20	22	2/20	NR
≥65	8/15	20	3/15	32

NR, not reached.

## Data Availability

The data presented in this study are available on request from the corresponding author. The data are not publicly available due to privacy restrictions.

## Abbreviations

The following abbreviations are used in this manuscript:

OC	Ovarian cancer
PFS	Progression-free survival
OS	Overall survival
ECOG	Eastern Cooperative Oncology Group
FIGO	International Federation of Gynecology and Obstetrics
VEGF	Vascular endothelial growth factor
